# Supplementary material for: Hydrogen evolution with hot electrons on a plasmonic-molecular catalyst hybrid system
Source: Nat Commun. 2024 Jan 10;15:445. doi: 10.1038/s41467-024-44752-y (PMC10781775; doi:10.1038/s41467-024-44752-y)
Supplement: Supplementary file 1 — Supplementary Information [file 41467_2024_44752_MOESM1_ESM.pdf]

Supplementary information

Hydrogen evolution with hot electrons on a plasmonic-molecular catalyst hybrid system

Ananta Dey[1], Amal Mendalz,[1]Anna Wach[2,3], Robert Bericat Vadell[1], Vitor R. Silveira[1], Paul Maurice Leidinger[2], Thomas Huthwelker[2], Vitalii Shtender[4], Zbynek Novotny[2], Luca Artiglia[2], Jacinto Sá[1,5]*

[1] Department of Chemistry-Ångström, Physical Chemistry division, Uppsala University, Box 532, 751 20 Uppsala, Sweden.

[2] Paul Scherrer Institut, CH-5232 Villigen PSI, Switzerland.

[3] SOLARIS National Synchrotron Radiation Centre, Jagiellonian University, Krakow, Poland.

[4] Department of Materials Science and Engineering, division of Applied Materials Science, Uppsala University, 75103 Uppsala, Sweden.

[5] Institute of Physical Chemistry, Polish Academy of Sciences, Marcina Kasprzaka 44/52, 01-224 Warsaw, Poland.

*Corresponding author. Email: jacinto.sa@kemi.uu.se

Sample preparation:

Au nanoparticles (NPs) synthesis:

The Au NPs were synthesised following the Turkevich method, as published by Piella et al.[1] Briefly, sodium citrate tribasic dihydrate (Merck, ACS reagent ≥ 99%) 50 mL (6.6 mM) water solution was taken in a 100 mL round bottom flask and stirred at 70 °C in an oil bath. Then, 0.1 mL (2.5 mM) tannic acid (Merck, ACS reagent ≥ 99.5%) was added to the reaction mixture. Finally, 1mL of (25 mM) $HAuCl_4$ (Merck, ≥ 99.9%) was added instantly. After 5 minutes, the reaction mixture changed from dark blue to wine. The colour change confirms the formation of the Au nanoparticles. The synthesised Au nanoparticles were stored in a fridge. The size of the Au nanoparticles was analysed using dynamic light scattering (DLS).

Cobalt catalyst synthesis:

200 mg (1.02 mmol) 1,10-Phenanthrolin-5-amine (Merck, 97%) and 148.42 mg (0.51 mmol) cobalt(II) nitrate hexahydrate (Merck, ACS reagent ≥ 98%) were refluxed in 20 ml ethanol (Merck, ≥ 99.9% (GC)) for one hour. After that, the reaction mixture was filtered. The filtrate was placed in a clean beaker at room temperature for a few days without further disturbance, and we got a red precipitate. Finally, the obtained product was collected through filtration and dried in a vacuum desiccator. The yield 216 mg (73.9%). ATR-IR 3342 cm^{-1}, 3402 cm^{-1}.

Preparation of the thin films:

<u>The preparation and process of NiO film:</u>

The NiO paste was purchased from Solaronix (Ni-Nanooxide N/SP, ~ 20 wt. %) and used as received. A small portion of the NiO paste was placed on a screen on top of the cleaned FTO glass and manually printed on the conducting side of the FTO glass. Then, the NiO-printed FTO glass plates were annealed at 500 °C for 1 hour at a rate of 10 °C/min.

<u>Assembly of the Au nanoparticles on NiO film:</u>

The synthesised Au nanoparticles were sprayed manually on NiO films and annealed at 500 °C for 1 hour at a rate of 10 °C/min.

<u>Assembly of the catalyst on NiO/Au surface:</u>

The annealed NiO-Au films were dipped into a 4 mg/mL water solution of the $[Co^{II}(phen\text{-}NH_2)_2(H_2O)_2]$ catalyst for 3 days. Finally, we could get the self-assembled NiO/Au/[Co^{II}(phen-

$NH_2)_2(H_2O)_2]$ composite system. The system was rinsed with water several times to remove unbounded catalyst molecules. For experimental purposes, we have sprayed Au nanoparticles only on FTO glass and on FTO/NiO and attached the molecular linker using the same procedure to deposit the catalyst.

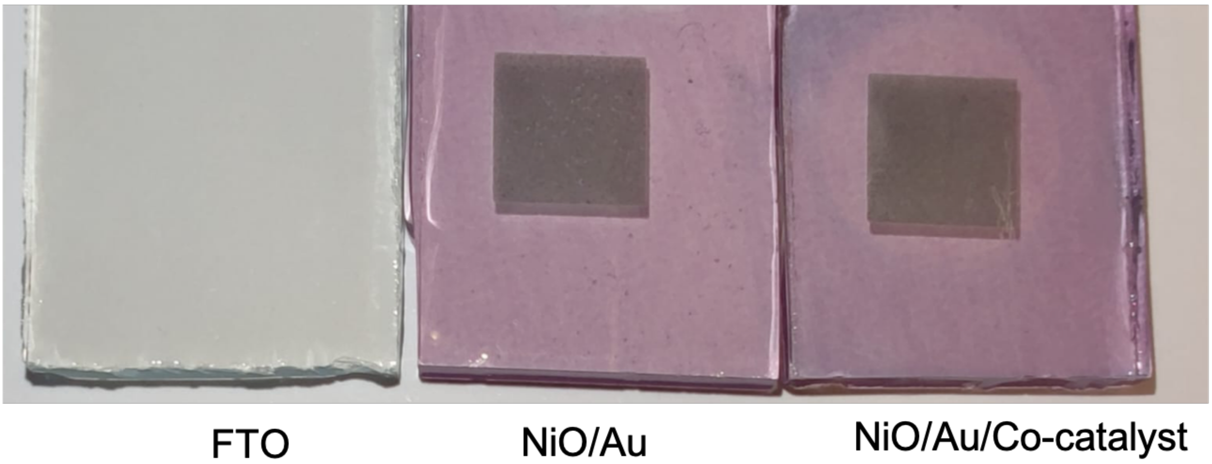

FIGURE S1: Photographs of the films prepared. The NiO is screen printed, and the Au NPs are sprayed across the entire glass to ensure coverage uniformity.

Samples characterisation:

UV-Vis measurements:

The UV-Vis spectra were collected using a Cary 5000 UV-VIS-NIR spectrophotometer.

Dynamic Light Scattering (DLS) measurements:

The DLS data was collected in a Malvern Zetasizer nanoS instrument, and a total of 3 measurements comprised of 12 scans each time was done.

Atomic Force Microscopy (AFM) measurements:

The AFM data was collected on AFM nanosurf with a long Si cantilever in tapping mode with Al reflex.

Ultra High Vacuum (UHV) X-ray photoelectron spectroscopy (XPS) measurements:

The UHV XPS data was collected on Quantera II from Physical Electronics. The effect of the electric charge was corrected by referencing the carbon peak (285 eV). The deconvolution of spectra was carried out using CasaXPS programs, in which a peak fitting was performed using Gaussian–Lorentzian peak shape and Shirley-type background subtraction.

Attenuated Total Reflection - Fourier-transformed infrared (ATR-FTIR) measurements:

FT-IR data were measured using a universal ATR sampling assembly on a VERTEX 70v instrument.

Inductively coupled plasma-optical emission spectrometry (ICP-OES) measurement:

The ICP-OES was used to estimate the cobalt amount in the sample. The film was digested in 4 mL of nitric acid (Nitric acid 65%, FisherScientific) for a couple of hours. The small probe was diluted 10 times with milliQ water containing 2% HNO_3 and filtered with 0.2 µm syringe filters (Whatman) before measurement. Avio 200 Scott/Cross-Flow Configuration was used for ICP measurements. A calibration curve was formed for the measurements using a Cobalt Calibration Standard (CPAchem). Concentrations of 0, 0.1, 1 and 10 ppm of the Co were used to create a 4-point linear regression. All measured values are within a *relative standard deviation* (RSD) of 2%.

Electrochemistry measurements:

The electrochemical data were measured using an EmStat potentiostat instrument. For the electrochemical experiments, a typical cylindrical closed cell was used. The FTO films were placed on the side of the cell so that it could face the light.

Bulk electrolysis: In the presence of 0.1 M LiCl (Merck, ACS reagent ≥ 99%) as a supporting electrolyte and glassy carbon as a working electrode, a Pt wire as a counter electrode, Ag/AgCl (3M KCl in water, Merck) as reference electrode was used for the bulk electrolysis experiment.

Photo-electrocatalytic chronoamperometry:

The photoelectrode exposed area to light is 0.79 cm^2. Plasmonic excitation was performed with a 532 nm laser of 43.8 mW/cm^2 intensity. Pt wire counter electrode and Ag/AgCl (3M KCl/water) reference electrode were purchased from Redox.me and used as received. Lithium chloride purchased from Merck was used as a supporting electrolyte without further purification.

Mass Spectrometry analysis:

We measured the gas produced during the photoelectrochemical reaction using a quadrupole mass spectrometer (QMS) (HPR 20) from Hiden Analytical. Continuous argon gas flow (15 mL/min) through the cylindrical electrochemical cell during the measurement. Before applying the electrochemical potential, we saturated the electrochemical cell with argon to get a stable argon signal. Argon, O_2, and H_2 were measured continuously with the SEM detector throughout the measurement.

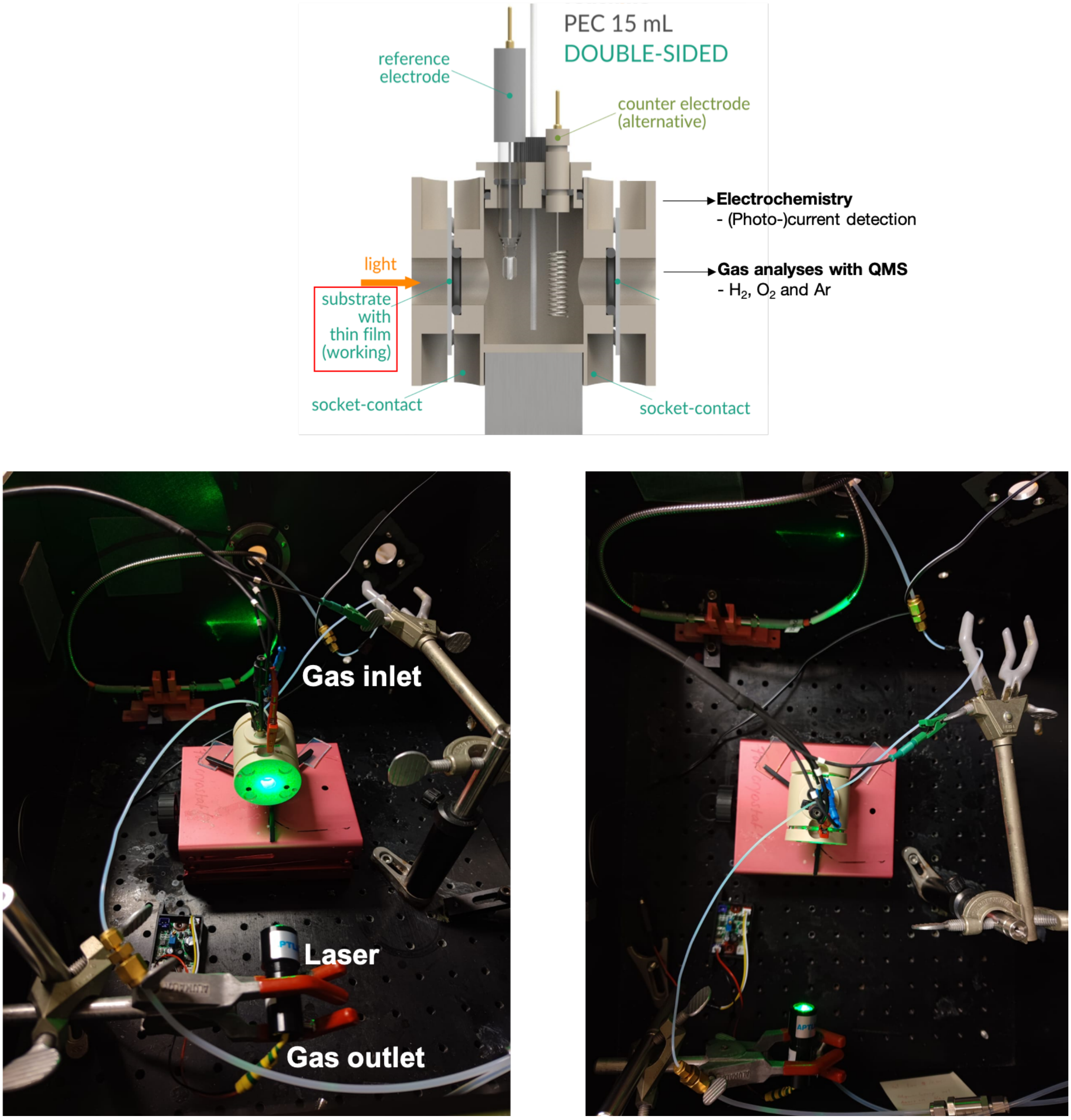

FIGURE S2: Photographs showing the photo-electrocatalytic chronoamperometry setup coupled with QMS. The gas outlet is connected to the QMS with a capillary, allowing flow measurements to occur.

Transient absorption spectroscopy (TAS)

A 40-fs pulsed laser with a 3 kHz repetition rate was generated through the Libra Ultrafast Amplifier System designed by Coherent. An optical parametric oscillator (TOPAS- prime, Light Conversion) created the excitation beam. The signals were detected with a UV-NIR detector from Newport MS260i spectrograph with interchangeable gratings. The fundamental laser (probe, 795 nm) passes through the delay stage (1-2 fs step size) and is focused in a

Sapphire optical window to generate visible light from 400 to 750 nm. The instrument response function obtained for our system is ca. 95 fs.

Transient infrared absorption spectroscopy (TIRAS)

A 40-fs pulsed laser with a 3 kHz repetition rate was generated through the Libra Ultrafast Amplifier System designed by Coherent. Two optical parametric oscillators (TOPAS- prime, Light Conversion) created the excitation beam and/or the probe light in the Mid-IR (3000-10000 nm). The signals were detected with a Horiba iHR 320 spectrometer. The pump laser power was constantly monitored with less than a 2% standard deviation. The timing resolution, i.e., the instrument response function, is ca. 100 fs.

Near-ambient pressure- X-ray photoelectron spectroscopy (NAP-XPS) measurements:

Details about the experimental chamber and the three-electrode setup have been described elsewhere.[2] NAP-XPS experiments were carried out at the PHOENIX I beamline of the Swiss Light Source Synchrotron (SLS), making use of the solid-liquid interface endstation in a three-electrode setup using a gold counter electrode and an Ag/AgCl reference electrode controlled via a potentiostat (BioLogic Science Instruments SP-300).[2] Linearly polarised light was used throughout the experiments. The as-introduced sample was first analysed under high vacuum conditions to acquire reference spectra. Then, the chamber was opened, and the beaker containing the pre-deaerated electrolyte was introduced (see Figure S1). The chamber was pumped down in a controlled way, using a needle valve, to avoid electrolyte spilling and favour the pressure equilibration (around 20 mbar). Measurements were carried out using an excitation energy of 5000 eV.

Deconvolution of the O *1s* spectra was performed after removing a Shirley background. Gaussian and Voigt-shaped components, whose positions were set according to past literature reports, were used to obtain the best correlation with experimental data (see Figure S15). Fitting parameters (peak positions, full width at half maximum –FWHM- and % of Lorentian-Gaussian) are summarised in Table S1.

TABLE S1: Parameters used to deconvolve O 1s spectra.

Position (eV)	FWHM	% L-G
529.50	1.75	0
531.00	1.85	25
532.68 – 532.75	1.75	0
534.80 – 535.25	0.90	0

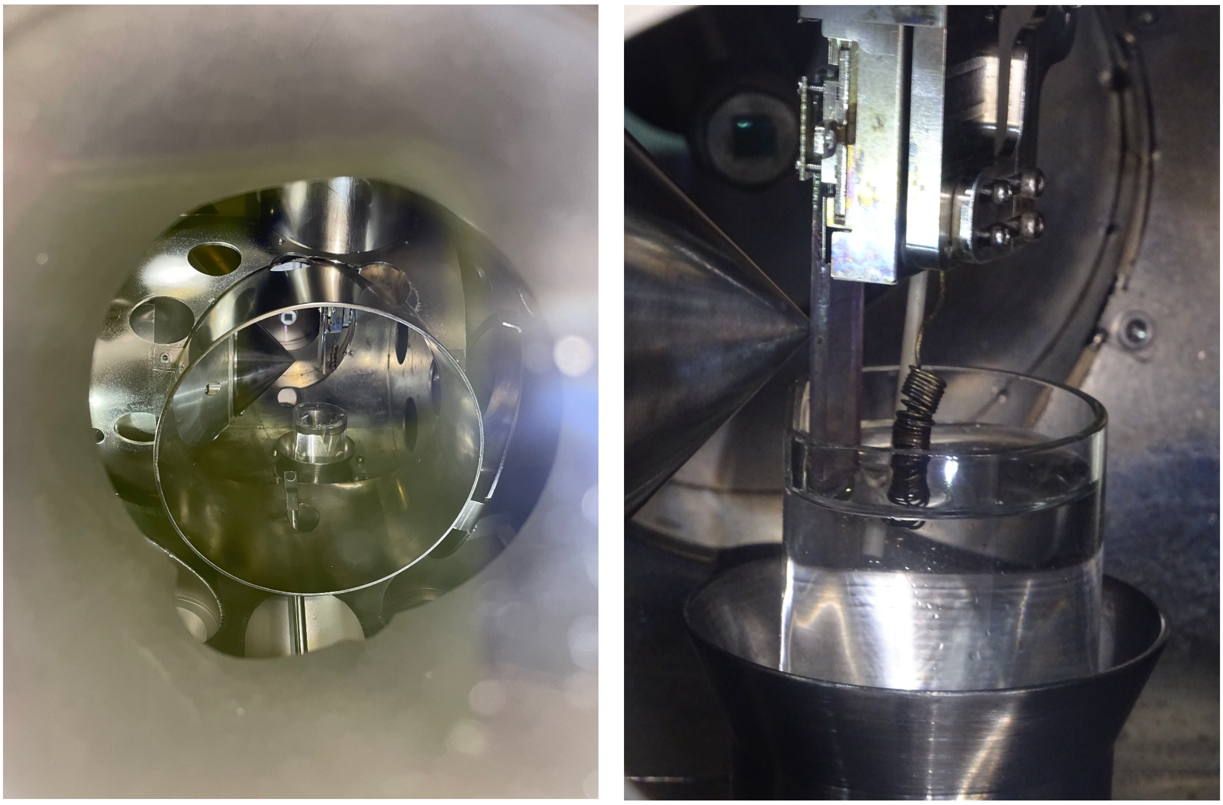

FIGURE S3: Photographs from inside the NAP-XPS chamber showing the beaker with electrolyte, working, reference and counter electrodes. The working electrode is the same system used for the chronoamperometry experiments, namely mesoporous film supported on FTO conductive glass.

Additional data:

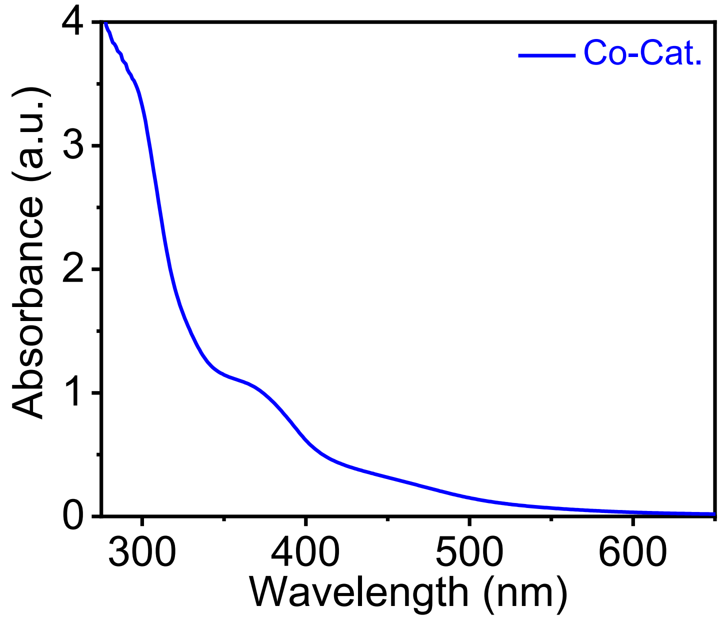

FIGURE S4: Optical spectrum of $[Co^{II}(phen\text{-}NH_2)_2NO_3]^{\cdot}NO_3^-$ in dimethylformamide.

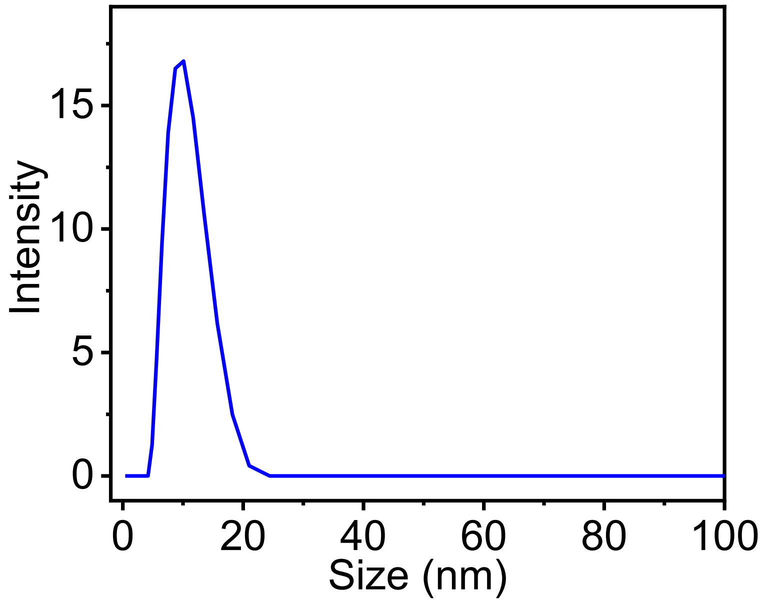

FIGURE S5: Dynamic light scattering (DLS) analysis of Au NPs in water.

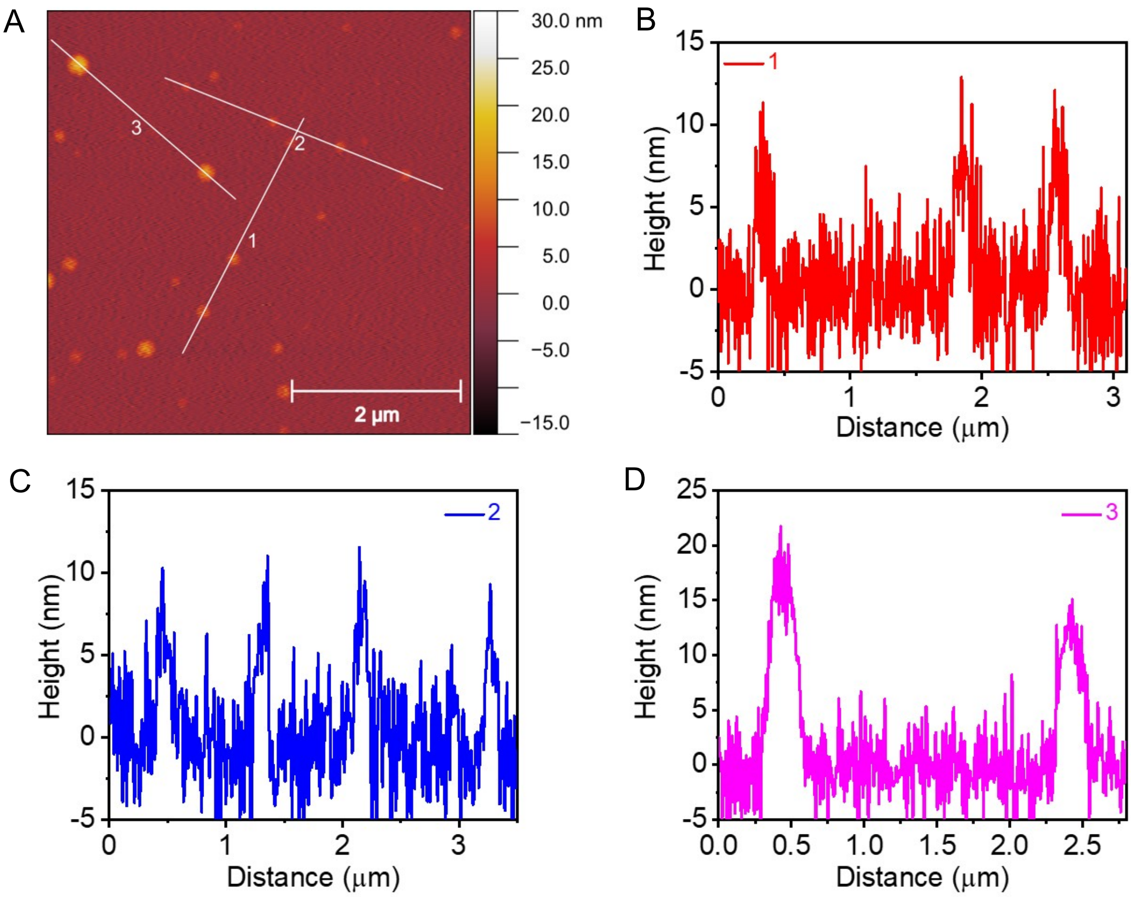

FIGURE S6: AFM of Au NPs on Si after annealing. A) AFM micrograph; and B), C) and D) show the line traces depicting the average particle height.

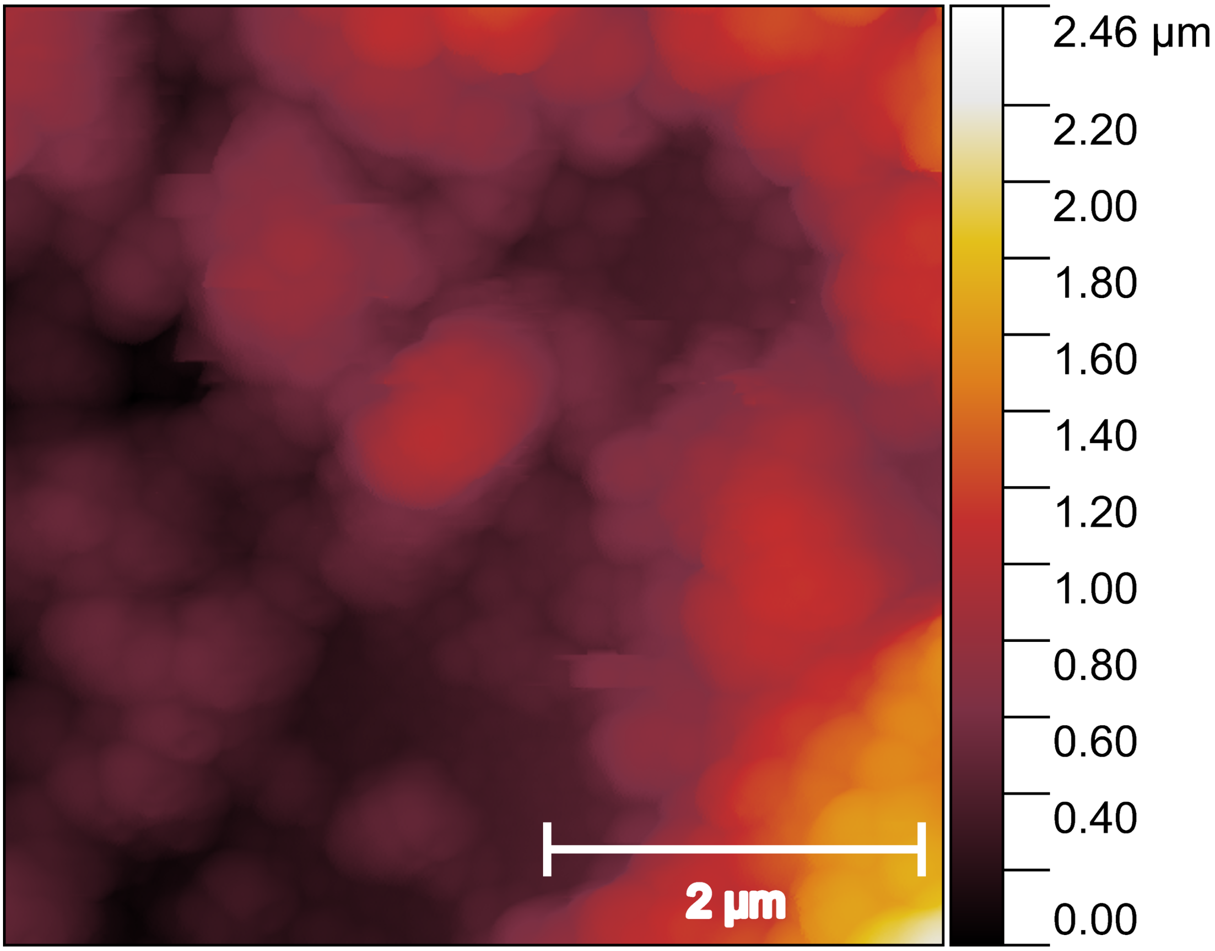

FIGURE S7: AFM micrograph showing NiO after depositing Au NP and second annealing at 500 °C for 1 hour. It is clear that the morphology of NiO film is kept including the porous, indicating that the second annealing does not affect film morphology.

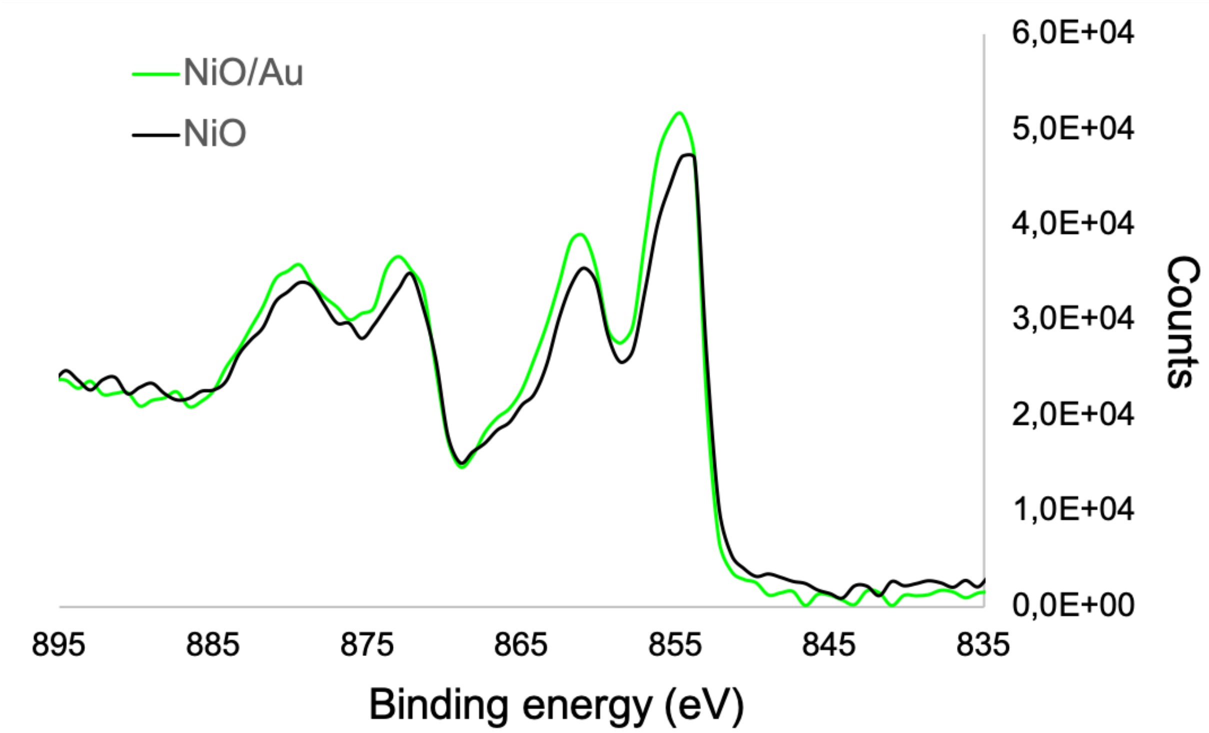

FIGURE S8: XPS of the Ni *2p* region of: A) NiO after annealing at 500 ºC for 1 hour; and B) NiO after depositing Au NP and second annealing at 500 ºC for 1 hour. The signal was acquired in the NAP chamber under low vacuum conditions.

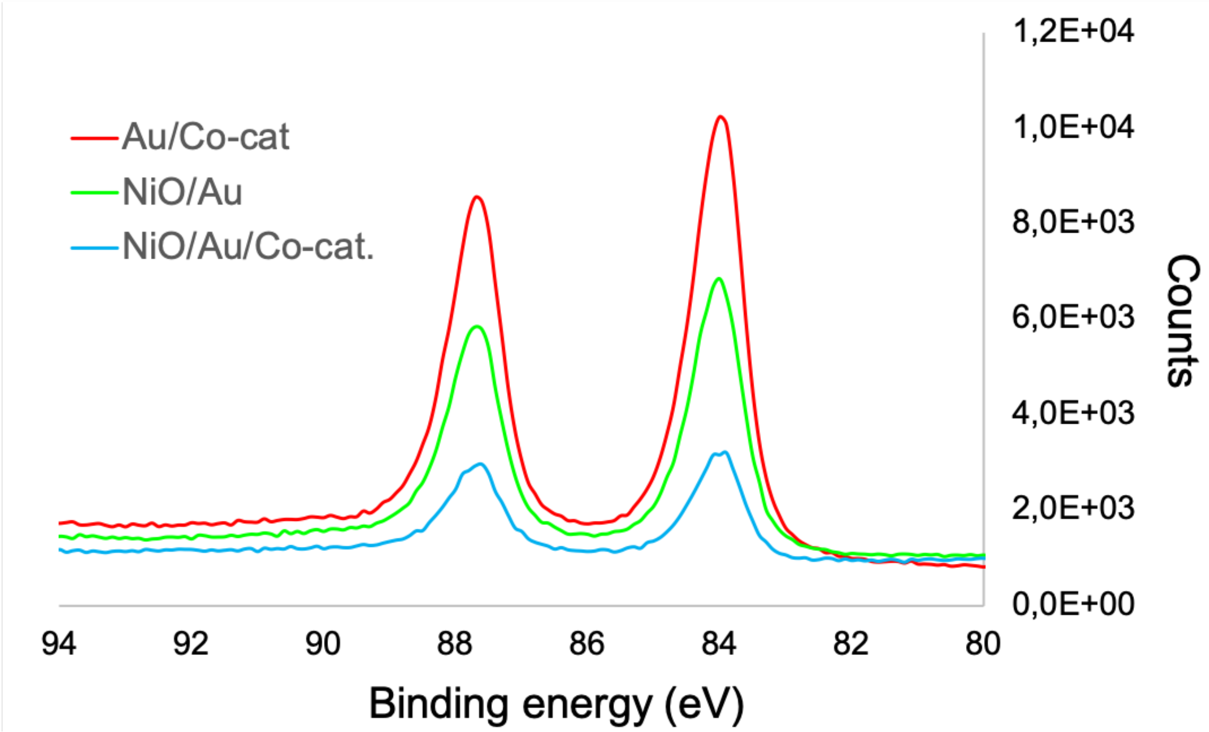

FIGURE S9: XPS of the Au *4f* region of Au/Co-cat, NiO/Au, and NiO/Au/Co-cat measured in the NAP chamber under low vacuum conditions.

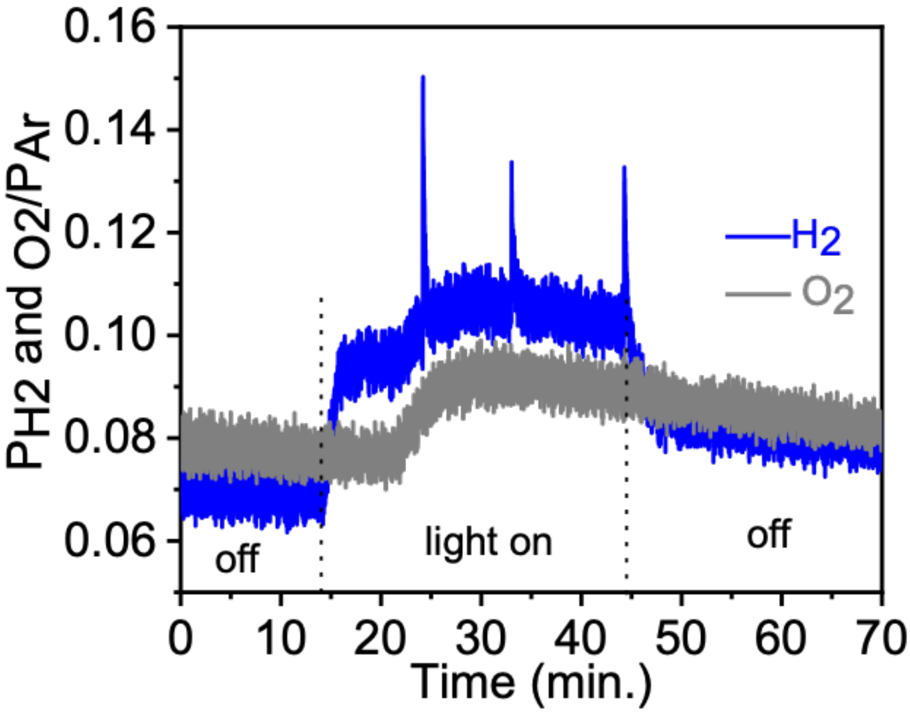

FIGURE S10: Online detection of H_2 and O_2 gases by QMS when applying -0.65V potential.

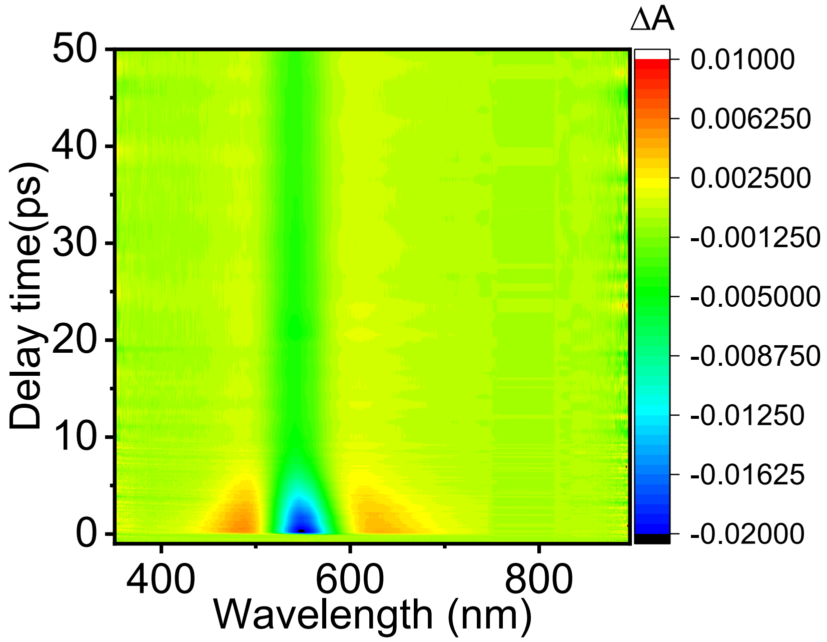

FIGURE S11: Representative contour-map plot of TAS data for Au NPs excited at 550 nm.

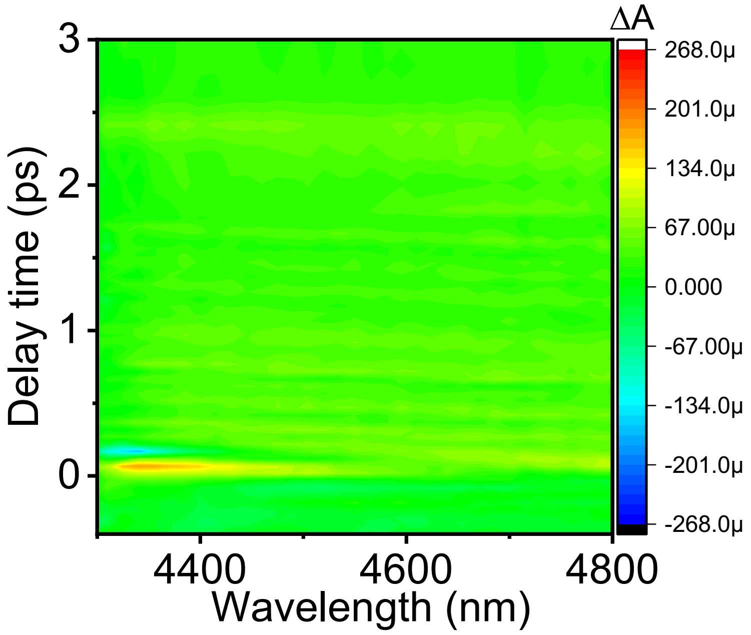

FIGURE S12: TIRAS contour plot of Au/Co-catalyst after excitation at 550 nm.

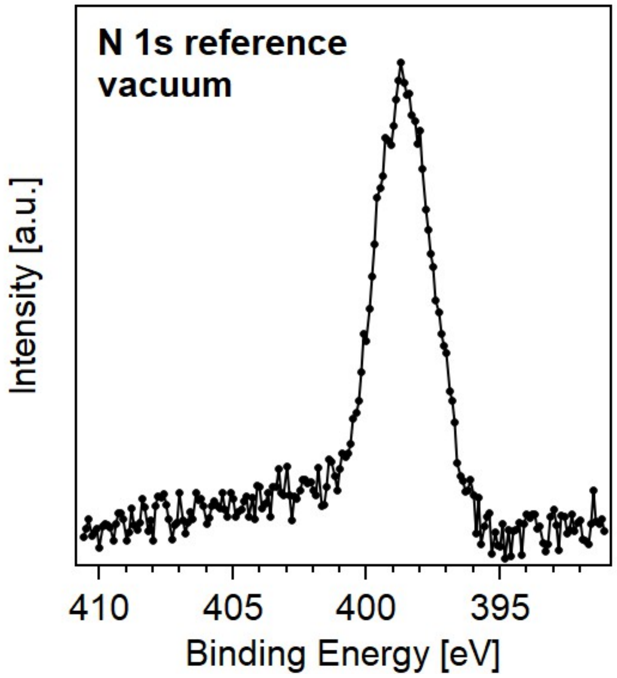

FIGURE S13: N *1s* photoemission spectrum of the "as-introduced" of the NiO/Au/Co-catalyst electrode acquired under vacuum.

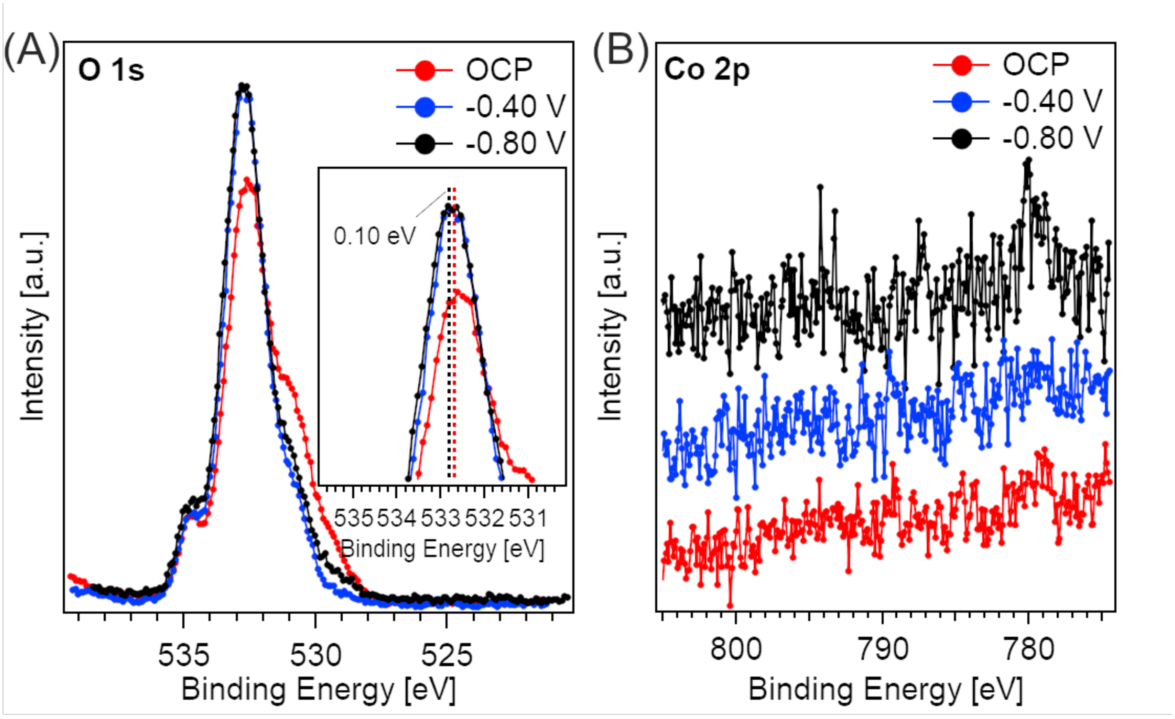

FIGURE S14: In situ NAP-XPS under the variable potential of the NiO/Au/Co-catalyst with an X-ray photon energy of 5000 eV without acid. A) O 1*s* signals (the inset magnifies the prominent peaks, highlighting the binding energy shift due to the potential applied); B) Co 2*p* signals.

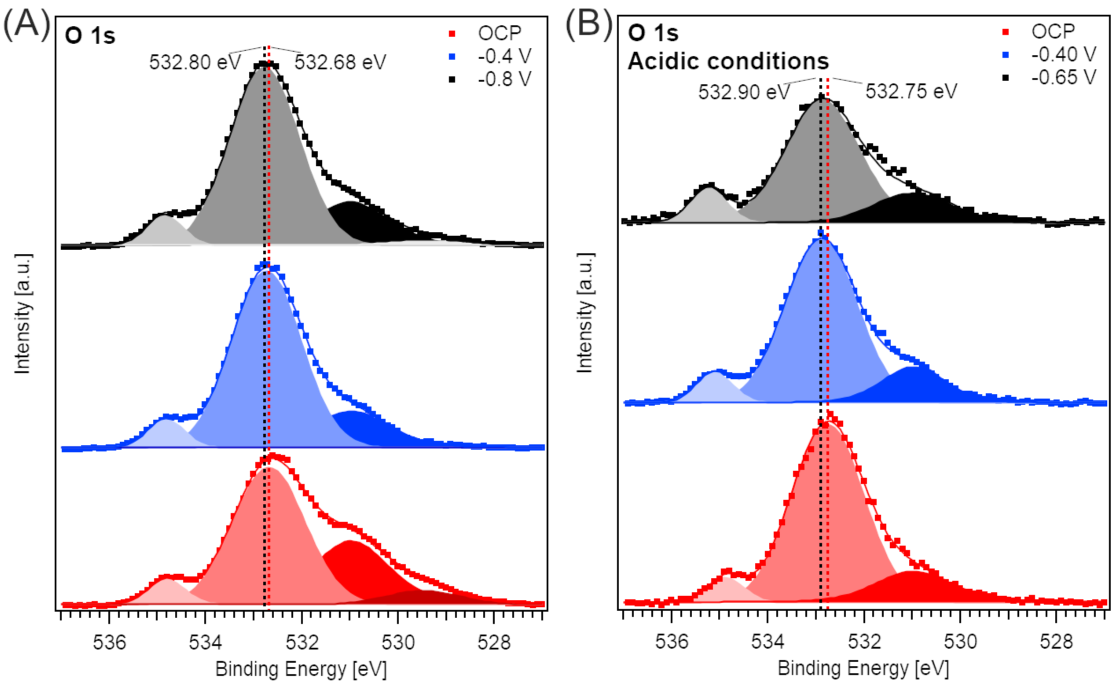

FIGURE S15: Deconvolution of the O *1s* NAP-XPS signals. A) without acetic acid in the electrolyte, and B) in the presence of 3 mM acetic acid (pH = 3.5) in the electrolyte.

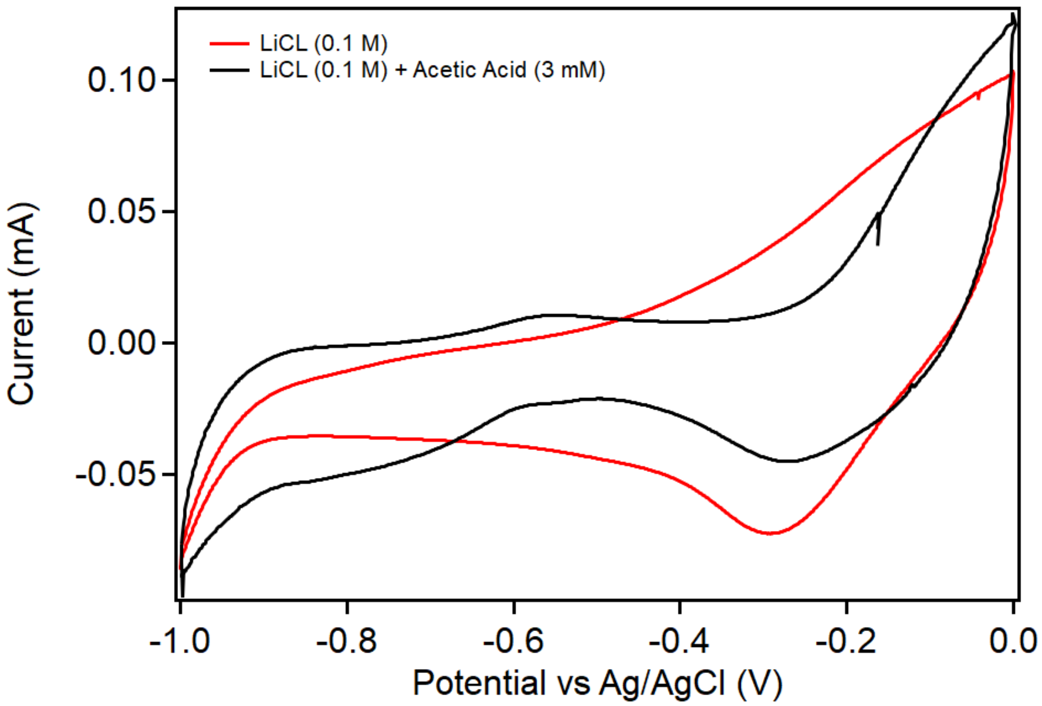

FIGURE S16: Representative cyclic voltammetry of the electrode in absence and presence of acid measured during the NAP-XPS measurements.

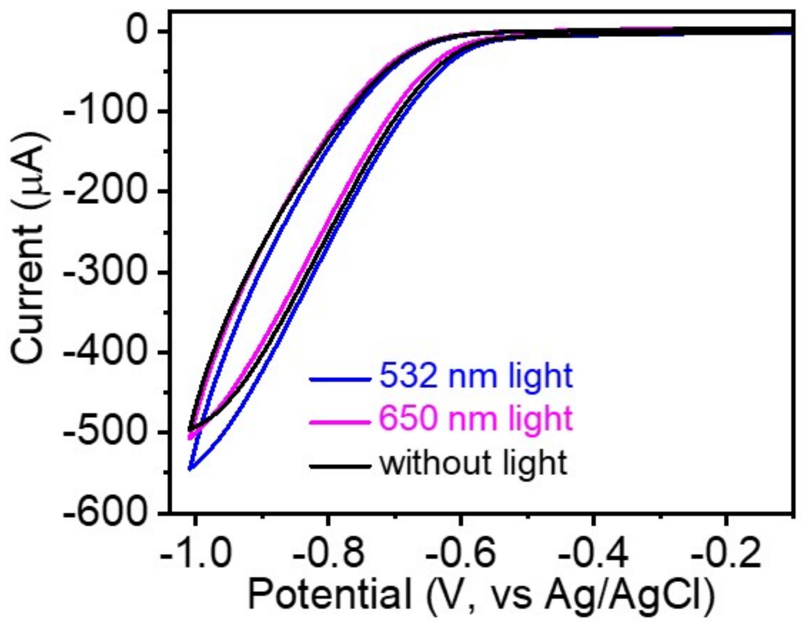

FIGURE S17: Representative cyclic voltammetry of the NiO/Au/Co-catalyst using 532 nm and 650 nm light in presence of 3 mM acetic acid and 0.1 M LiCl and Ag/AgCl as reference electrode. For comparison purposes, the CV from bulk electrolysis (without light) is also provided.

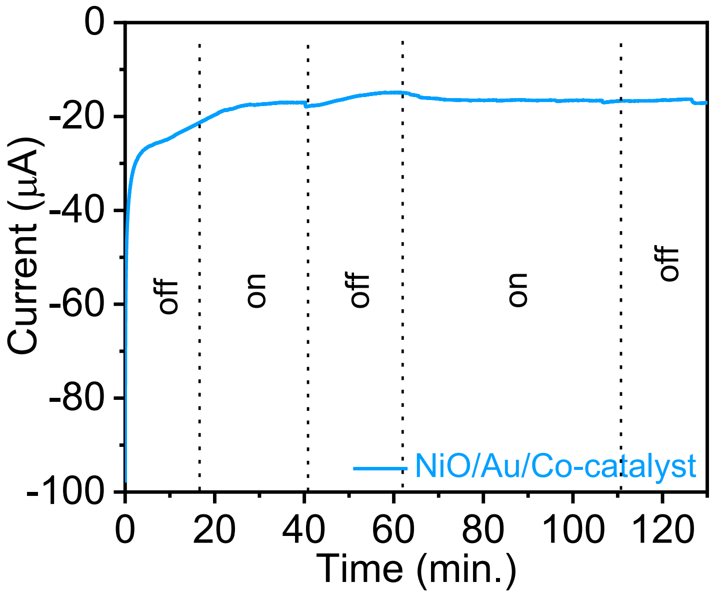

FIGURE S18: Representative chronoamperometry of the NiO/Au/Co-catalyst using 650 nm light in the presence of 3 mM acetic acid and 0.1 M LiCl and Ag/AgCl as reference electrode.

Supplementary References:

1. Piella, J., Bastús, N. G., Puntes, V. Size-Controlled Synthesis of Sub-10-nanometer Citrate-Stabilized Gold Nanoparticles and Related Optical Properties. *Chem. Mater*. **28**, 1066-5463 (2016).

2. Novotny, Z., Aegerter, D., Comini, N., Tobler, B., Artiglia, L., Maier, U., Moehl, T., Fabbri, E., Huthwelker, T., Schmidt, T., Ammann, M., van Bokhoven, J. A., Raabe, J., Osterwalder, J. Probing the solid–liquid interface with tender x rays: A new ambient-pressure x-ray photoelectron spectroscopy endstation at the Swiss Light Source. Rev. Scie. Instrum. 91, 023103 (2020).
